# Development and Validation of a Chiral Liquid Chromatographic Assay for Enantiomeric Separation and Quantification of Verapamil in Rat Plasma: Stereoselective Pharmacokinetic Application

**DOI:** 10.3390/molecules26072091

**Published:** 2021-04-06

**Authors:** Mostafa S. Mohammed, Mohamed M. Hefnawy, Abdulrhman A. Al-Majed, Haitham K. Alrabiah, Nasser A. Algrain, Ahmad J. Obaidullah, Abdulmalik S. Altamimi, Yousef A. Bin Jardan, Abdullah M. Al-Hossaini

**Affiliations:** 1 Department of Pharmaceutical Chemistry, College of Pharmacy, King Saud University, P.O. Box 2457, Riyadh 11451, Saudi Arabia; mostafanodcar@yahoo.com (M.S.M.); almajed99@yahoo.com (A.A.A.-M.); halrabiah@ksu.edu.sa (H.K.A.); n_algrain@hotmail.com (N.A.A.); aobaidullah@ksu.edu.sa (A.J.O.); abalhossaini@ksu.edu.sa (A.M.A.-H.); 2 National Organization for Drug Control and Research, Cairo P.O. Box 29, Egypt; 3 Department of Analytical Chemistry, Faculty of Pharmacy, Mansoura University, Mansoura 35516, Egypt; 4 Department of Pharmaceutical Chemistry, College of Pharmacy, Prince Sattam Bin Abdulaziz University, Al-Kharj 11942, Saudi Arabia; as.altamimi@psau.edu.sa; 5 Department of Pharmaceutics, College of Pharmacy, King Saud University, P.O. Box 2457, Riyadh 11451, Saudi Arabia; ybinjardan@ksu.edu.sa

**Keywords:** verapamil enantiomers, core–shell chiral column, chiral HPLC, rat plasma, stereoselective pharmacokinetics

## Abstract

A novel, fast and sensitive enantioselective HPLC assay with a new core–shell isopropyl carbamate cyclofructan 6 (superficially porous particle, SPP) chiral column (LarihcShell-P, LSP) was developed and validated for the enantiomeric separation and quantification of verapamil (VER) in rat plasma. The polar organic mobile phase composed of acetonitrile/methanol/trifluoroacetic acid/triethylamine (98:2:0.05: 0.025, *v/v/v/v*) and a flow rate of 0.5 mL/min was applied. Fluorescence detection set at excitation/emission wavelengths 280/313 nm was used and the whole analysis process was within 3.5 min, which is 10-fold lower than the previous reported HPLC methods in the literature. Propranolol was selected as the internal standard. The S-(−)- and R-(+)-VER enantiomers with the IS were extracted from rat plasma by utilizing Waters Oasis HLB C18 solid phase extraction cartridges without interference from endogenous compounds. The developed assay was validated following the US-FDA guidelines over the concentration range of 1–450 ng/mL (r^2^ ≥ 0.997) for each enantiomer (plasma) and the lower limit of quantification was 1 ng/mL for both isomers. The intra- and inter-day precisions were not more than 11.6% and the recoveries of S-(−)- and R-(+)-VER at all quality control levels ranged from 92.3% to 98.2%. The developed approach was successfully applied to the stereoselective pharmacokinetic study of VER enantiomers after oral administration of 10 mg/kg racemic VER to Wistar rats. It was found that S-(−)-VER established higher C_max_ and area under the concentration-time curve (AUC) values than the R-(+)-enantiomer. The newly developed approach is the first chiral HPLC for the enantiomeric separation and quantification of verapamil utilizing a core–shell isopropyl carbamate cyclofructan 6 chiral column in rat plasma within 3.5 min after solid phase extraction (SPE).

## 1. Introduction

Fast and efficient separation of a wide range of drugs is a challenge in HPLC. Fast separation results in high operating pressure, which places a massive load on HPLC instrumentation [1]. Furthermore, higher selectivity and sensitivity are advantages of using UHPLC in analytical chromatography [2]. Unfortunately, the use of UHPLC is counteracted by some of its limitations such as low peak area repeatability values due to the small injection volumes, high difficulty in packing of the tiny sub-2 µm particle columns, and the short column lifetime, which may be attributed to the high back pressure [3,4]. In recent years, core–shell silica particles stationary phases (solid core and porous shell) have been used as an alternative technology to overcome these disadvantages due to the highly efficient separation with a fast flow rate and reasonably low back pressure [5]. The porous shell and small solid core can afford a higher surface area for the separation to occur while the solid core plus the porous shell gives a larger particle and thus low operating back pressure [5]. Studies comparing the conventional totally porous particles and core–shell particles have been done to determine different drug groups [6,7,8,9,10]. There are few studies on the application of core–shell silica materials in chiral chromatography [11,12,13]. Recently, isopropyl cyclofructan-6 bonded superficially porous particles (SPPs) were shown to afford higher efficiency and faster separations for pharmaceutical, biological and forensic samples [14,15].

**Figure 1:**
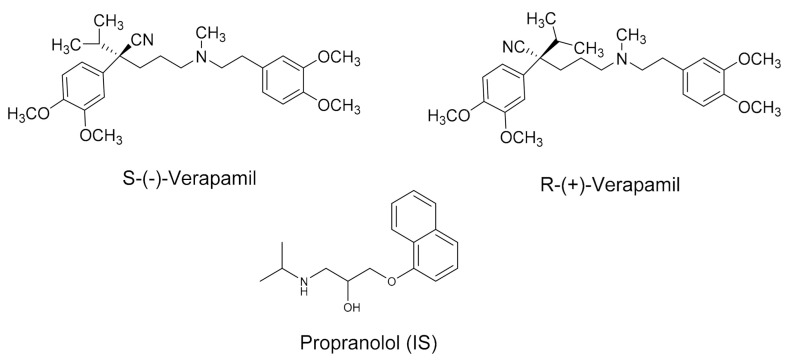
Chemical structure of verapamil enantiomers and propranolol (IS).

Verapamil (VER, 2-(3,4-dimethoxyphenyl)-5-[2-(3,4-dimethoxyphenyl)ethyl-methylamino]-2-propan-2-yl-pentanenitrile, Figure 1), is a phenylalkylamine calcium channel blocking drug used in the treatment of heart arrhythmias, high blood pressure and angina. VER was the first calcium channel antagonist to be introduced into therapy, and works by relaxing the muscles of the heart and blood vessels [16]. VER is obtainable as a racemate (rac-VER) that contains equal amounts of S-(−)- and R-(+)-isomer. The two isomers are readily distinguished by biological systems, they have different pharmacokinetic behavior, pharmacodynamics and/or toxicological effects [17]. In previous studies conducted in humans, the S-(−)-enantiomer carries approximately 20-fold greater potency than the R-(+)-enantiomer in slowing cardiac conduction velocity [18]. Moreover, in contrast to human plasma, the effect of S-(−)- and R-(+)-enantiomers of VER on rat plasma was different in vivo [19]. The toxic level of VER is observed at concentrations of more than 1000 ng/mL, while the therapeutic plasma concentration of the drug ranged from 20 to 500 ng/mL [20]. Therefore, a therapeutic drug monitoring (TDM) study is essential due to its narrow therapeutic index [21]. Moreover, enantiomeric analysis of racemic drugs should be considered to both maximize the efficacy and minimize the toxicity of drug therapy [22].

There are several bioanalytical reported methods for chiral resolution and determination of VER using HPLC [23,24,25,26,27,28,29,30,31,32,33,34,35,36], capillary electrophoresis (CE) [37,38,39,40,41,42,43] and LC–MS/MS [44,45,46,47,48,49]. CE methods display excellent chiral separation; however, the CE assay needs a longer run time of approximately 32 min for enantioseparation [40,41]. Moreover, the CE technique is not always available in clinical laboratories [50]. Although LC-MS/MS is a potential tool in determination of drugs, its high cost and instrumentation complexity limit its routine application in bioanalytical laboratories. For enantiomeric separation and quantification of racemic drugs, HPLC is preferred due to high inherent specificity, high accuracy and remarkable precision with low cost. However, the literature reveals that reported HPLC methods for enantioanalysis of VER use a high amount of organic solvents with a longer analysis time of approximately 34–40 min [23,30,31]. The chiral separation HPLC bioanalytical methods of VER that have been previously reported are summarized in Table 1. The alternative approach is the application of core–shell isopropyl carbamate cyclofructan 6 chiral stationary phase (LarihcShell-P, LSP) for highly efficient separation with reduced run times (3.5 min). This core-shell chiral column provides the same efficient separations as the sub 2 μm particles that are used in UHPLC without the disadvantage (high back pressure).

In this study, a rapid, sensitive and enantioselective chiral HPLC-FL method was developed for determination of S-(−)- and R-(+)-VER utilizing a core–shell (superficially porous particle, SPP) isopropyl carbamate cyclofructan 6 chiral column in rat plasma after SPE, for the first time. The established assay exhibited excellent performance in terms of selectivity and efficiency (3.5 min) with a simple sample preparation. The validated assay employs a very low plasma volume (50 µL) for processing and has a sensitivity (1.0 ng/mL) 20-fold lower than other reported HPLC methods for enantiomeric determination of VER [30,31]. This assay was fully validated and successfully applied in a stereoselective pharmacokinetic study in rats after oral administration of VER racemate.

## 2. Results and Discussion

### 2.1. Method Development and Optimization

Superficially porous particles (core–shell) chiral stationary phases have revealed advantages in enantiomeric separations in HPLC owing to their superior effective separation with significantly reduced analysis times [4]. The polar organic mobile phase (POM) has been described as a mode to attain enantioselective resolution with derivatized CF-CSPs. This mode uses a non-aqueous polar solvent (acetonitrile) with both acetic acid and triethylamine, which are required to achieve difficult enantioseparation [12]. This study aimed to develop a novel chiral bioanalytical HPLC assay for the fast and sensitive determination of VER enantiomers, permitting a low level sensitivity (1.0 ng/mL) in plasma and ability to being used in stereoselective pharmacokinetic studies of VER in rats.

In previous HPLC studies, the chiral separations of VER enantiomers were achieved using α_1_-acid glycoprotein (AGP), cellulose tris(3,5-di-methylphenylcarbamate (Chiralcel OD-R) and amylose tris(3,5-di-methylphenylcarbamate (Chiralpak AD) CSPs that required a relatively long run time of approximately 34–40 min to attain acceptable chiral separation [23,30,31]. In this study, an effective alternative HPLC method using core shell isopropyl cabamate cyclofructan 6, LarihcShell-P, (LSP) CSP was established to provide higher efficiency and shorter analysis time to improve the throughput analysis.

The preliminary investigations were directed to use a conventional HPLC technique with three different fully porous derivatized CF-CSP columns and three different mobile phase modes to acquire an optimized separation and symmetrical peak shape for VER enantiomers and IS. The three fully porous derivatized CF-CSP columns included dimethylphenyl carbamate cyclofructan 7 (Larihc CF7-DMP), R-naphthylethyl carbamate cyclofructan 6 (Larihc CF6-RN) and isopropyl carbamate cyclofructan 6 (Larihc CF6-P) (150 mm × 4.6 mm i.d, 5 µm particle size) from AZYP, LLC. (Arlington, TX, USA). The screening mobile phase compositions were performed for these columns with normal phase mode (NP), reversed phase mode (RP) and polar organic mode (POM). The NP screening mobile phase consisted of heptane/ethanol/triethylamine (TEA) (70:30:0.1, *v/v/v*) and an acetonitrile (ACN)/acetate buffer (pH 4.0, 20 mM) (70:30, *v/v*) for RP. The POM screening mobile phase consisted of ACN/methanol (MeOH)/trifluoroacetic acid (TFA)/TEA (80:20:0.1:0.1, *v/v/v/v*), examined at a flow rate of 0.5 mL/min. To assess when to use each solvent adjustment, the parameters that impact resolution (Rs) (retention factor (*k*), selectivity (α), and efficiency (N)) were carefully studied and compared [51]. Subsequently, α was first targeted to increase Rs, α has a greater influence on Rs. As a whole, parameters like temperature and flow rate did not significantly increase α but they affected N and *k.* Consequently, a variety of mobile phase optimization factors such as acid-base ratio, salt concentration and an organic modifier were examined to improve Rs.

**Figure 2:**
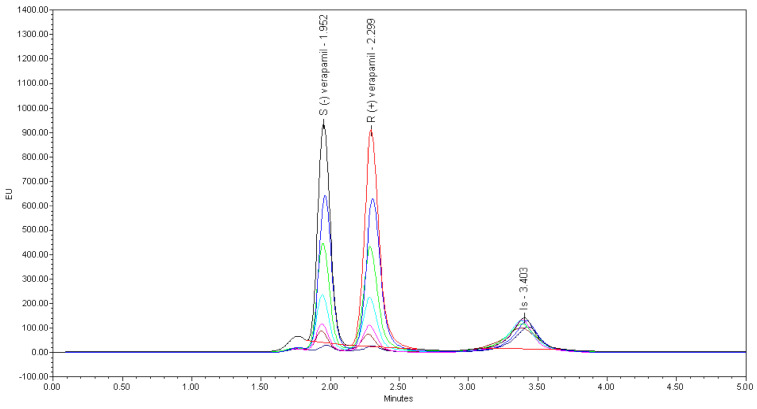
Representative overlaid HPLC chromatograms of the rat plasma analysis of S-(−)-verapamil (1.95 min), R-(+)-verapamil (2.29 min) (1.0–450 ng/mL) and propranolol (3.38), (IS), (500 ng/mL).

The separation of VER enantiomers was first attempted using CF7-DMP, CF6-RN and CF6-P columns with the above mentioned three mobile phase compositions. However, no chiral resolution was achieved on CF6-RN and CF7-DMP columns, but partial separation of VER enantiomers (Rs = 0.9) was observed on a CF6-P column with a mobile phase consisted of ACN/MOH/TFA/TEA (98:2:0.1:0.1, *v/v/v*). However, the tailing factors of the enantiomers were so large that a good peak could not be obtained, and the retention time was longer than 18 min. Moreover, when the ratio of TFA/TEA became 0.05:0.025, (*v/v*), the resolution of VER enantiomers improved (Rs = 1.1), but the retention time was still longer (18 min). An effective baseline separation of VER enantiomers (Rs = 2.82), separation factor (α > 1.15) and improved peak shape of the VER enantiomers with a short analysis time (2.3 min) were attained when the core–shell (superficially porous particle, SPP), LarihcShell-P (LSP) (100 mm × 2.1 mm i.d., 2.7 μm particles) CSP, with an isocratic mobile phase consisting of ACN/MOH/TFA/TEA (98:2:0.05: 0.025, *v/v/v/v*) at a flow rate of 0.5 mL/min was applied. An adequate chiral chromatography separation with well-defined peaks for S-(−)-, R-(+)-VER, and IS in rat plasma was achieved with retention times of 1.95, 2.29, and 3.38 min for S-(−)-, R-(+)-VER, and IS; respectively, as shown in Figure 2 and Table 2.

### 2.2. Sample Pretreatment

Sample pretreatment is critical for determination of analytes. Liquid–liquid extraction (LLE) and solid phase extraction (SPE) are common methods used for sample pretreatment [52]. LLE could not effectively remove the interferences caused by endogenous substances from the sample matrix, and it could not afford clean enough samples. In the current study, SPE was a useful extraction procedure related to LLE, which has been reported in the previous HPLC studies [23,25,26,27,31,33], where it provided less solvent consumption, was less time-consuming, and had low background noise and high recoveries of the S-(−)-, R-(+)-VER and IS from rat plasma. Five types of SPE cartridges (Water oasis HLB, CN, C18, C8 and C2) were examined for plasma sample cleanup. The eluting capabilities of numerous solvents toward VER enantiomers and IS were studied. Of these solvents, only MeOH rather than a mixture of MeOH with water or ACN was able to disrupt all types of interactions in the case of VER enantiomers and IS and thus to extract them from the C18 sorbent. High recoveries and clean chromatogram for S-(−)-, R-(+)-VER and IS were attained with the C18 cartridge. The recoveries ranged from 92.3–98.2 and 92.0–97.9 for S-(−)- and R-(+)-VER in the rat plasma, respectively. In the present study, propranolol was selected as IS because its physicochemical characteristics are similar to those of VER and it gave high recovery and good separate peaks from the S-(−)- and R-(+)-VER. Both drugs possess native fluorescence, similar solubilities in alcohols, and nearly similar log *p* values.

### 2.3. In-Study Validation

According to the guidelines of the US-FDA [53], method validation parameters, such as specificity, calibration curves, lower limit of quantification (LLOQ), intra- and inter-day accuracy and precision, extraction recovery and stability were evaluated.

**Figure 3:**
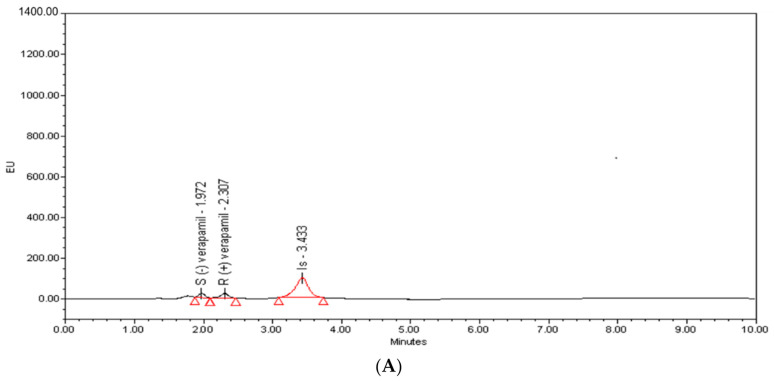

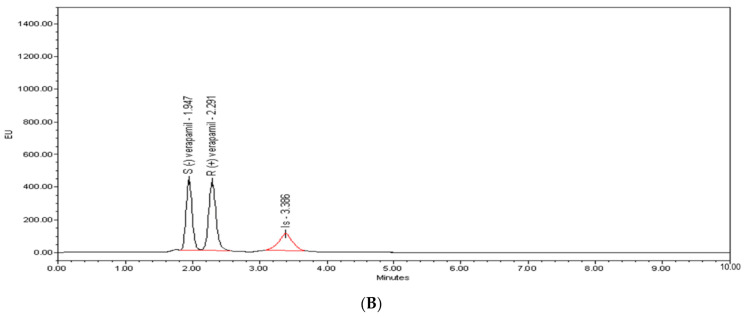
Representative HPLC chromatograms of blank plasma spiked with S-(−)- and R-(+)-verapamil at LLOQ level (**A**) and a plasma sample obtained at 2 h after a single dose of 10 mg/kg racemic verapamil (**B**).

The specificity of the developed HPLC-FL method was evaluated by comparing the chromatograms of blank plasma samples, the plasma samples spiked with VER enantiomers at LLOQ (1 ng/mL) for each enantiomer and real plasma samples collected at 2 h after oral administration of 10 mg/kg racemic VER. The typical chromatograms are shown in Figure 3B. The retention time of S-(−)-, R-(+)-VER, and IS were 1.95, 2.29, and 3.38 min, respectively. No interfering peaks of endogenous components of the rat plasma were observed at the retention time of VER enantiomers and IS, which confirms that the validated method is specified.

Calibration curves for S-(−)-, R-(+)-VER enantiomers were validated in the concentration range of 1.0–450.0 ng/mL in rat plasma. The calibration curve was constructed by plotting the peak area ratio (*y*) of each enantiomer to the IS versus the spiking concentrations (*x*) of each enantiomer. The regression equations achieved by least squared regression for S-(−)- and R-(+)-VER were; y = 0.0014x + 0.0121, r^2^ = 0.998; and y = 0.0018x + 0.0192, r^2^ = 0.997, for S-(−)- and R-(+)-VER, respectively (Table 3). The LLOQ of each VER enantiomer was recognized as 1 ng/mL. These results confirmed that the developed assay was proper for the research on the enantioselective pharmacokinetics of VER in rat.

The intra- and inter-day precision and accuracy values for QC samples (LLOQ, LQC, MQC, HQC) of S-(−)- and R-(+)-VER are summarized in Table 4. Precision was determined by the relative standard deviation (RSD), while the relative error (RE) was employed to estimate the accuracy. The RSD values of intra- and inter-day precision for S-(−)-VER were <11.7%, whereas the RE values of accuracy vary from −1.0 to 5.3%. For R-(+)-VER, the RSD values of intra- and inter-day precision were within 9.8%, while the RE values of accuracy ranged from −3.1 to 3.6%. At LLOQ, the RSD for both intra- and inter-assay precision was not more than 10.2% and 8.2% for S-(−)- and R-(+)-VER; respectively. These data demonstrated that the optimized assay revealed satisfactory precision and accuracy.

The extraction recoveries were assessed by comparing the peak areas obtained from pre-extracted plasma samples spiked with S-(−)- and R-(+)-VER and post-extracted plasma samples spiked with analytes. The recoveries of S-(−)- and R-(+)-VER at three QC levels (3, 200 and 400 ng/mL) and IS (500 ng/mL) ranged from 92.3% to 98.2% and from 92.0% to 97% with all RSD values within 9.5% (Table 5). The extraction recoveries of IS at 500 µg/mL in plasma were >94%.

Stability of the S-(−)- and R-(+)-VER enantiomers in rat plasma at the two different concentration levels (LQC, HQC) during the sample storing and processing procedures was fully assessed and the results are summarized in Table 6. The data of stability tests showed that S-(−)- and R-(+)-VER were stable at room temperature for 24 h (short-term stability) and at −80 °C for 30 days (long-term stability). To examine autosampler stability, samples were retained in an automatic sampling room at 10 °C for 24 h. For freeze-thaw stability, samples were frozen at −80 °C for 24 h and then thawed to room temperature for three cycles. As per the stability conditions, the accuracy in plasma ranged from −6.2% to 2.9% and −7.6% to 4.4% for S-(−)- and R-(+)-VER, respectively. The results confirmed that the VER enantiomers maintained stability in plasma samples under different storage conditions.

### 2.4. Application to an Enantioselective Pharmacokinetic Study

**Figure 4:**
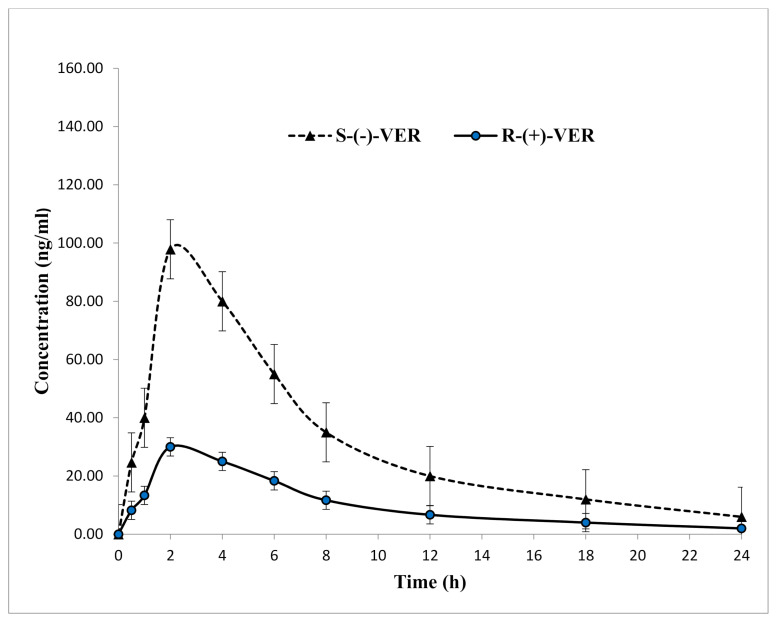
Mean plasma concentration-time profile of S-(−)- and R-(+)-verapamil after oral (gavage) administration of 10 mg/kg racemic verapamil hydrochloride to Sprague-Dawley rats, (*n* = 6, mean ± SD).

The applicability of the developed method in this study was established through the determination of S-(−)- and R-(+)-VER in rat plasma after oral (gavage) administration of 10 mg/kg racemic verapamil [54]. The typical chromatograms of rat plasma at 2.0 h after oral administration are shown in Figure 3B. The curve of mean plasma concentration over time of VER enantiomers is presented in Figure 4, and the individual differences in the pharmacokinetic (PK) parameters of the S-(−)- and R-(+)-VER are summarized in Table 7. Differences in PK parameters were considered statistically significant at *p* < 0.05, by comparing paired sample T-tests. By comparing the drug concentration-time curves of the S-(−)- and R-(+)-VER more precisely, we found that the mean C_max_ value of S-(−)-VER was 3.2 times higher than that of R-(+)-VER, and the area under the plasma concentration versus time curve (AUC_0–∞_) was 3-fold higher than that of R-(+)-VER, which repeatedly verified that S-(−)-VER offered the greater pharmacodynamic activity compared with R-(+)-VER. These results are consistent with in vivo rac-verapamil PK data from previous studies [19,55] in which the S-isomer form of VER is almost totally responsible for the slowing cardiac conduction velocity. Moreover, the plasma concentration of S-(−)-VER after oral administration had always been much higher than that of R-(+)-VER, which might be beneficial if S-(−)-VER performs the pharmacodynamic action in vivo. This study justified the conclusion that the pharmacokinetics of the VER enantiomers after oral administration of racemic VER were stereoselective. The use of rats in this work instead of humans was because of the significant correlation between the lipoprotein lipid and protein profiles in humans and rats [56].

## 3. Materials and Methods

### 3.1. Chemicals and Reagents

S-(−)-, R-(+)-verapamil and racemic-verapamil hydrochloride (99.0%, purity) were purchased from Toronto Research Chemicals (Toronto, ON, Canada). Propranolol (internal standard, IS, 98.0% purity) was obtained from Sigma-Aldrich (St. Louis, MO, USA). HPLC-grade methanol and acetonitrile LiChrosolv^®^ (99.0% purity) were from Merck (Darmstadt, Germany). Trifluoroacetic acid and triethylamine (98% purity) were purchased from Sigma-Aldrich (Zwijndrecht, The Netherlands). All other chemicals and solvents were obtained from BDH Chemicals (Poole, UK). The water used was purified by a Milli-Q Millipore Water System (Millipore, Billerica, MA, USA). Oasis HLB and Sep-Pak cartridges CN, C18, C8 and C2 (1 mL) were obtained from Waters Corp. (Milford, MA, USA).

### 3.2. Chromatographic Conditions

Chromatographic analysis was achieved on a Waters Corporation System (Milford, MA, USA), equipped with a binary pump (Waters 1525), an autosampler (Waters 2707), a dual wavelength fluorescence detector (Waters 2475) and an Empower Pro Chromatography Manager software (Waters Corporation) for data acquisition. The signal was monitored at 280 nm for excitation and 313 nm for emission. The chiral chromatographic separation was carried out at 21 ± 2 °C on an isopropyl carbamate cyclofructan 6 (superficially porous particle, SPP) analytical chiral column (LarihcShell-P, LSP) (100 mm × 2.1 mm i.d., 2.7 μm particles), purchased from AZYP, LLC. (Arlington, TX, USA) with a guard column of LarihcShell-P, LSP (3.0 mm i.d. × 10 mm, 2.7 μm particles, AZYP, Arlington, TX, USA). The mobile phase was acetonitrile/methanol/trifluoroacetic acid/triethylamine (98:2:0.05: 0.025, *v/v/v/v*) at a flow rate of 0.5 mL/min. The injection volume was 5.0 μL and total run time was 3.5 min. The mobile phases and drug solutions were filtered through a 0.22 µm Millex filter (EMD Millipore, Milford, MA, USA) and degassed in an ultrasonic bath (Tecnal, São Paulo, Brazil). Three fully porous derivatized CF-CSP columns from AZYP (Arlington, TX, USA) with the following characterization: 150 mm × 4.6 mm i.d, 5 µm particle size, were used for a comparison study of R-naphthylethyl carbamate cyclofructan 6 (Larihc CF6-RN), isopropyl carbamate cyclofructan 6 (Larihc CF6-P) and dimethylphenyl carbamate cyclofructan 7 (Larihc CF7-DMP).

### 3.3. Animals

Male Sprague-Dawley rats (250–290 g) were procured from the Animal Care Center of King Saud University (Riyadh, Saudia Arabia), and were utilized to gather blank plasma and to conduct the pharmacokinetic studies. The blank plasma was obtained by centrifuging drug-free rat blood for 10 min at 2500× *g*, then storing it at −80 °C until analysis. All the animal experimental procedures involving animal handling were carried out according to the ethical guidelines for experimental studies with animals according to the Institutional Animal Care and Use Committee (IACUC) guidelines, King Saud University, with ethical approval number KSU-SE-19-11.

### 3.4. Sample Preparation

#### 3.4.1. Preparation of Stock and Standard Solutions

The methanol was used for preparing the 1 mg/mL stock solutions of the S-(−)- and R-(+)-VER (free base) and propranolol (IS). Working solutions of the S-(−)- and R-(+)-VER at concentrations of 0.1, 1, and 10 µg/mL (intermediate solutions) were prepared by diluting its stock solutions with methanol. The working solution of IS (500 ng/mL) was achieved by diluting the stock solution with methanol. All standard solutions were stored at −20 °C.

#### 3.4.2. Preparation of Calibrators and Quality Control Samples

Calibrators at concentrations of 1, 5, 10, 50, 100, 250 and 450 ng/mL for S-(−)- and R-(+)-VER were prepared in drug-free rat plasma from the intermediate solutions. Quality control (QC) samples at low (3 ng/mL), medium (200 ng/mL), and high (400 ng/mL) blank plasma concentrations of VER enantiomer were prepared.

### 3.5. Sample Pretreatment

Plasma (50 µL) in a 2 mL disposable polypropylene micro centrifuge tube containing 1 mL of phosphate buffer (0.05 mM, pH 9.0) was spiked with 50 μL of propranolol (IS) (500 ng/mL). The tube was twisted for 30 s. Oasis HLB (Waters) cartridges were attached to a vacuum manifold (VacElute, Harbor City, CA, USA) and conditioned with 2 × 500 µL of methanol and 2 × 500 µL of deionized water. Care was taken to assure that the cartridges did not dry out. The buffered blank and plasma sample was loaded into the cartridges and a vacuum was applied to gain a flow rate of 0.5 mL/min. The cartridges were washed with 2 × 500 µL of deionized water, then dried under pressure (15 psi) for 3 min. The analytes were eluted with 2 × 100 µL of methanol and 5 µL was injected into the HPLC system.

### 3.6. Pre-Study Validation

Intensive validation studies for analyzing S-(−)-, R-(+)-VER in rat plasma were performed following the US-FDA guidelines [53]. The studied validation parameters in the rat plasma included specificity, linearity and sensitivity, accuracy and precision, recovery, influence of co-elution of enantiomers and stability.

The specificity of the developed HPLC-FL method was examined by evaluating the interference levels of endogenous components in blank rat plasma from six individuals. Levels of less than 20% of the LLOQ for S-(−)-, R-(+)-VER and <5% of the IS were accepted [53].

The linearity of the calibration curves in plasma, described as y = a + bx, was established using seven concentrations of S-(−)- and R-(+)-VER enantiomer; 1, 5, 10, 50, 100, 250 and 450 ng/mL, on three separate days. The lowest concentration of the calibration curve (S/N ≥ 10) was utilized for determining the LLOQ.

Intra- and inter-day precision and accuracy were assessed by assaying six replicates of spiked plasma samples at the lower limit (LLOQ) (1 ng/mL), in addition to three different QC levels (3, 200, and 400 ng/mL) for each enantiomer, on three successive days (inter-day, n = 3) and one day (intra-day, n = 3). The accuracy was expressed as RE (%) and the precision as RSD (%). The impact of the co-elution of S-(−)-VER and R-(+)-VER on the method values was assessed by subjecting plasma samples containing high levels of S-(−)-VER and low levels of R-(+)-VER (or vice versa) to the analysis.

The extraction recovery of S-(−)- and R-(+)-VER enantiomers in plasma was examined by comparing the peak areas obtained from blank plasma samples spiked with the analytes before extraction with those of spiked post-extraction at three QC levels (LQC, MQC, and HQC) in six replicates. Moreover, the extraction recovery of the IS at the same concentration level of the method was calculated.

The stability of S-(−)-VER and R-(+)-VER in rat plasma was evaluated using QC samples at LQC, MQC, and HQC in three replicates. The QC samples were kept at room temperature for 24 h and at −80 °C for 30 days to evaluate the short-and long-term stability, respectively. The freeze-thaw stability examination was investigated after exposing the unextracted QC samples to three freeze-thaw cycles (3 cycles, −80 °C). The post preparative stability was tested in an autosampler at 10 °C for 24 h.

### 3.7. Stereoselective Pharmacokinetic Studies

The validated HPLC-FL method was used to investigate the stereoselective pharmacokinetic (PK) study of VER enantiomers in rats. Six male Sprague-Dawley rats (250–290 g) were fasted for 12 h before the experiment, while water was allowed ad libitum. On the day of experiments, rats were treated by gavage administration with a single oral dose of 10 mg/kg rac-verapamil hydrochloride dissolved in 0.9% saline [54]. Blood samples (300 μL) were collected into tubes containing di-sodium EDTA at the following time points: 0 (before administration), 0.5, 1, 2, 4, 6, 8, 12, 18 and 24 h. The plasma was separated and collected by centrifuging blood at 2500× *g* for 10 min and transferred to pre-labeled Eppendorf tubes and kept at −80 °C until analysis. The PK parameters were calculated by fitting the data to a noncompartmental analysis (NCA) model with PK Solver Add-In software [57].

## 4. Conclusions

In this study, a rapid, sensitive and accurate HPLC-FL method was developed to analyze verapamil enantiomers in rat plasma after SPE for the first time. The established assay provided enough separation for verapamil enantiomers using a new core–shell isopropyl carbamate cyclofructan 6 (superficially porous particle, SPP) chiral column (LarihcShell-P, LSP) in the polar organic mode. The LLOQ of each verapamil enantiomer was established to be 1 ng/mL, which was significantly lower than the previous report [30,31] (20 ng/mL). The whole analysis process was within 3.5 min, which is 10-fold lower than the previous reported HPLC methods in the literature [23,30,31]. After validation, the developed method was applied to the enantioselective pharmacokinetic study of verapamil enantiomers after oral administration of 10 mg/kg racemic verapamil to Wistar rats. It was found that AUC, C_max_, and clearance values of S-(−)-verapamil were prominently higher than those of R-(+)-verapamil, indicating the enantioselective pharmacokinetic behavior of verapamil in rats.

## Figures and Tables

**Table 1:** Chiral HPLC separation methods reported for verapamil determination.

Determinate Method	Chiral Selector	Composition of the Mobile Phase	Matrix	Ext. Method	Run Times (min)	LR (ng/mL)	Ref.
HPLC-Fluorescence	Chiral-AGP column (α_1_-acid glycoprotein) (100 mm × 4.0 mm, 5 µm).	Acetonitrile: 10 mM sodium perchlorate, pH 7.0 (10:90, *v/v*)	Human serum	SPE	40	20–400	[30]
HPLC-Fluorescence	Chiral-AGP column (α_1_-acid glycoprotein) (100 mm × 4.0 mm, 5 µm).	Acetonitrile: 10 mM sodium perchlorate, pH 7.0 (10:90, *v/v*)	Human serum	LLE	40	20–400	[31]
HPLC-Fluorescence	Chiralcel OD-RH column (cellulose tris(3,5-di- methylphenylcarbamate) (150 mm × 4.6 mm, 5 µm)	Acetonitrile: 30 mM hexafluorophosphate, pH 4.6 (34:66, *v/v*)	Human plasma	LLE	34	10–250	[23]
HPLC-Fluorescence	Chiralcel OD-RH column (cellulose tris(3,5-di- methylphenylcarbamate) (250 mm × 4.6 mm, 10 µm)	Acetonitrile: 0.2 M sodium perchlorate (40:60, *v/v*)	Urine	SPE	25	2.5–300	[24]
HPLC-Fluorescence	Chiralpak AD column (amylose tris(3,5-di- methylphenylcarbamate) (250 mm × 4.6 mm, 5 µm)	*n*-hexane: isopropanol: diethylamine (94:6:0.1) (*v/v/v*)	Rat plasma and tissues	LLE	20	100–500	[25]
HPLC-Fluorescence	Chiralpak AD column (amylose tris(3,5-di- methylphenylcarbamate) (250 mm × 4.6 mm, 5 µm	*n*-hexane: isopropanol: diethylamine (90:10:0.1) (*v/v/v*)	Human plasma	LLE	20	50–500	[26]
HPLC-Fluorescence	Chiralpak AD column (amylose tris(3,5-di- methylphenylcarbamate) (250 mm × 4.6 mm, 5 µm	*n*-hexane: isopropanol: triethylamine (85:15:0.4) (*v/v/v*)	Human plasma	LLE	20	2.5–100	[27]
HPLC-Fluorescence	Chiral-AGP column (α_1_-acid glycoprotein) (100 mm × 4.0 mm, 5 µm).	Acetonitrile: 30 mM phosphate buffer, pH 5.3 (4:96, *v/v*)	Human serum	LLE	20	10–120	[33]

AGP: Alpha1-acid glycoprotein; AD: amylose tris(3,5-di- methylphenylcarbamate; OD-RH: cellulose tris(3,5-di- methylphenylcarbamate; HPLC: High-performance liquid chromatography; SPE: olid phase exteaction; LLE: Liquid–liquid extraction; LR: Linear rang.

**Table 2:** Chromatographic parameters of verapamil enantiomers and internal standard in rat plasma using the proposed method.

Analyte	R_s_^a^	α ^b^	k ^c^	T_R_ (min) ^d,e^
S-(−)-verapamil	f	1.24	4.21 ± 0.03	1.95 ± 0.04
R-(+)-verapamil	2.82	1.75	5.22 ± 0.08	2.29 ± 0.07
Propranolol (IS)	8.68	^f^	9.13 ± 0.04	3.38 ± 0.04

^a^ R_s_ = 2 (t_2_ − t_1_)/(w_1_ + w_2_), where t_2_ and t_1_ are the retention of the second and first peaks and w_1_ and w_2_ are the half peak width of the second and first peaks. ^b^ Separation factor, calculated as k_2_/k_1_. ^c^ Retention capacity. ^d^ T_R_ = Retention time. ^e^ (mean ± SD). ^f^ Not calculated.

**Table 3:** Statistical parameters of calibration curves for S-(−)- and R-(+)-verapamil in rat plasma using the developed method.

Parameters	S-(−)-Verapamil	R-(+)-Verapamil
Concentration range (ng/mL)	1–450	1–450
Intercept (a)	1.21 × 10^−2^	1.91 × 10^−2^
Slope (b)	1.45 × 10^−3^	1.81 × 10^−3^
Coefficient of determination (r2)	0.998	0.997
S Y/N ^a^	7.39 × 10^−3^	6.88 × 10^−3^
Sa ^b^	4.52 × 10^−3^	3.29 × 10^−3^
Sb ^c^	2.74 × 10^−3^	5.14 × 10^−3^
LLOQ (ng/mL)	1.0	1.0
LLOD (ng/mL)	0.3	0.3

^a^ SD of the residual. ^b^ SD of the intercept. ^c^ SD of the slope.

**Table 4:** Precision and accuracy data for analysis of verapamil enantiomers in rat plasma (3 days with 6 replicates per day).

Analyte				Intra-Day			Inter-Day	
		Nominal Conc. (ng/mL)	Mean (ng/mL)	Precision (RSD, %)	Accuracy (RE, %)	Mean (ng/mL)	Precision (RSD, %)	Accuracy (RE, %)
S-(−)- verapamil	LLOQ	1	1.03 ± 0.10	9.72	3.00	1.04 ± 0.08	7.69	4.00
	QCL	3	3.14 ± 0.33	10.41	4.66	3.16 ± 0.36	11.61	5.33
	QCM	200	197.00 ± 7.3	3.82	−1.50	198.00 ± 7.54	3.81	−1.00
	QCH	400	390.00 ± 8.55	2.19	−2.50	387.08 ± 12.04	3.11	−3.23
R-(+)- verapamil	LLOQ	1	1.04 ± 0.11	10.19	4.00	1.05 ± 0.09	8.14	5.00
	QCL	3	3.11 ± 0.28	9.15	3.66	3.09 ± 0.30	9.82	3.00
	QCM	200	197.22 ± 8.30	4.21	1.39	196.50 ± 7.29	3.71	1.75
	QCH	400	387.60 ± 11.43	2.95	−3.10	390.68 ± 10.97	2.81	−2.33

**Table 5:** Extraction recovery for the analysis of S-(−)- and R-(+)-verapamil and IS in rat plasma by the proposed method.

Nominal Concentration (ng/mL)	S-(−)-Verapamil			R-(+)-Verapamil			IS
	3	200	400	3	200	400	500 (ng/mL)
Mean ^a^	2.77	191.50	392.88	2.76	193.70	391.64	470.55
RSD	9.51	4.22	2.71	7.19	3.82	2.83	8.91
Recovery (%)	92.33	95.75	98.22	92.00	96.85	97.91	94.70
Mean recovery (%)	95.58			94.92			94.11

^a^ mean of six measurements.

**Table 6:** Stability results for S-(−)- and R-(+)-verapamil in rat plasma under different storage conditions.

Analyte	Concentration (ng/mL)		Short Term Stability at Room Temperature (24 h)		Freeze and Thaw Stability at −80 °C (3 cycles)		Long Term Stability at −80 °C (30 days)		Autosampler Stability at 10 °C (24 h)	
			RE (%)	RSD (%)	RE (%)	RSD (%)	RE (%)	RSD (%)	RE (%)	RSD (%)
S-(−)-verapamil	QCL	3	−5.11	9,70	−6.22	11.40	−4.82	11.31	−5.21	9.29
	QCH	400	1.42	1.81	2.63	6.52	2.91	3.85	0.75	3.24
R-(+)-verapamil	QCL	3	−4.73	10.61	−7.61	11.52	−5.35	5.52	−3.82	5.29
	QCH	400	1.29	2.76	4.34	6.35	2.52	4.45	3.73	4.44
N			3		3		3		3	

**Table 7:** The enantioselective pharmacokinetic parameters of S-(−)- and R-(+)-verapamil in the plasma of rats treated with a single dose of 10 mg/kg racemic verapamil hydrochloride (gavage), (*n* = 5, Mean ± SD).

Parameter	Unit	S-(−)-VER *	R-(+)-VER *
AUC0-t ^a^	ng/mL·h	754.10 ± 150.82	244.10 ± 48.82
AUC0-∞ ^b^	ng/mL·h	810.30 ± 162.95	262.82 ± 52.51
*C*max ^c^	ng/mL	97.85 ± 19.62	30.25 ± 6.43
*T*max ^d^	h	2.00 ± 0.41	2.00 ± 0.41
Cl/F^e^	ng/mL.h	12.34 ± 2.47	38.05 ± 7.61
t_1/2_^f^	h	6.50 ± 1.30	6.50 ± 1.33
MRT_0-∞_^g^	h	8.97 ± 1.80	9.14 ± 1.83

* Data are presented as mean ± SD. ^a^ Area under the concentration-time curve to the last measurable concentration. ^b^ Area under the plasma concentration-time curve to infinity. ^c^ Maximum plasma concentration. ^d^ Time to reach maximum plasma concentration. ^e^ Total body clearance. ^f^ Elimination half-life. ^g^ Mean residence time.

## Data Availability

Not applicable.

## Sample Availability

Samples of the compounds are not available from the authors.
